# A Filamentous Hemagglutinin-Like Protein of *Xanthomonas axonopodis* pv. *citri*, the Phytopathogen Responsible for Citrus Canker, Is Involved in Bacterial Virulence

**DOI:** 10.1371/journal.pone.0004358

**Published:** 2009-02-04

**Authors:** Natalia Gottig, Betiana S. Garavaglia, Cecilia G. Garofalo, Elena G. Orellano, Jorgelina Ottado

**Affiliations:** Molecular Biology Division, Instituto de Biología Molecular y Celular de Rosario, Consejo Nacional de Investigaciones Científicas y Técnicas, Facultad de Ciencias Bioquímicas y Farmacéuticas, Universidad Nacional de Rosario, Rosario, Argentina; Massachusetts General Hospital, United States of America

## Abstract

*Xanthomonas axonopodis* pv. *citri*, the phytopathogen responsible for citrus canker has a number of protein secretion systems and among them, at least one type V protein secretion system belonging to the two-partner secretion pathway. This system is mainly associated to the translocation of large proteins such as adhesins to the outer membrane of several pathogens. *Xanthomonas axonopodis* pv. *citri* possess a filamentous hemagglutinin-like protein in close vicinity to its putative transporter protein, XacFhaB and XacFhaC, respectively. Expression analysis indicated that *XacFhaB* was induced *in planta* during plant-pathogen interaction. By mutation analysis of *XacFhaB* and *XacFhaC* genes we determined that *XacFhaB* is involved in virulence both in epiphytic and wound inoculations, displaying more dispersed and fewer canker lesions. Unexpectedly, the *XacFhaC* mutant in the transporter protein produced an intermediate virulence phenotype resembling wild type infection, suggesting that XacFhaB could be secreted by another partner different from XacFhaC. Moreover, *XacFhaB* mutants showed a general lack of adhesion and were affected in leaf surface attachment and biofilm formation. In agreement with the *in planta* phenotype, adhesin lacking cells moved faster in swarming plates. Since no hyperflagellation phenotype was observed in this bacteria, the faster movement may be attributed to the lack of cell-to-cell aggregation. Moreover, *XacFhaB* mutants secreted more exopolysaccharide that in turn may facilitate its motility. Our results suggest that this hemagglutinin-like protein is required for tissue colonization being mainly involved in surface attachment and biofilm formation, and that plant tissue attachment and cell-to-cell aggregation are dependent on the coordinated action of adhesin molecules and exopolysaccharides.

## Introduction

Protein secretion in gram-negative bacteria is accomplished by different pathways that are highly conserved in bacterial species. Among them, the type V secretion system stands out by its apparent simplicity and comprises two distinct pathways, the autotransporter and the two-partner secretion (TPS) pathways [1]. Both, the autotransporter and TPS systems translocate large proteins or protein domains, mostly adhesins and hemolysins, and have been identified in many bacterial genera, including human, animal and plant pathogens. TPS systems are composed of two proteins, the transported protein named TpsA and the specific transporter TpsB that localizes in the outer membrane [2]. TpsA proteins share a highly conserved N-proximal region of approximately 250 residues essential for secretion called the ‘TPS domain’ [3], [4] that directs the secretion of the entire protein through the channel-forming outer membrane porin-like protein TpsB [2].

The well studied filamentous hemagglutinin (FHA) protein of the whooping cough agent *Bordetella pertussis* is a 230-kDa adhesin, named FhaB, is secreted by the other TPS partner FhaC, standing for TpsA and TpsB, respectively [5]. FhaB, as well as the other proteins secreted by the TPS pathway, contains the characteristic TPS domain in its N-proximal region whose structure has been determined. The domain core is folded into a right-handed parallel β-helix of nine coils that comprises three helical β–sheets [4]. Sequence comparison of this domain from different animal and plant pathogens revealed two conserved regions (C) and two less conserved regions (LC) in an alternating organization LC1–C1–LC2–C2 [4]. Since crystallographic analysis of the complete mature protein has not been possible due to the large size of FhaB, a truncated variant has been studied by electron microscopy. The results suggested that the most likely model for the mature protein would be that of an elongated β-helix in which the β-helical fold continues beyond the TPS domain [6].

Bacterial attachment is a crucial early step in pathogenesis. Specialized surface exposed proteins called adhesins mediate bacterial adhesion to accomplish this critical step. Numerous studies on adhesins from animal bacterial pathogens have indicated that they are required for optimal virulence. In plant pathogens the role of these proteins in virulence is less clear since it has been not analyzed in detail. A mutant in a gene codifying for the 3,850-aa protein homolog to FhaB from *B. pertussis* in *Erwinia chrysanthemi*, a pathogen that causes soft-rot on a wide variety of plants reduces its ability to attach to and form aggregates on *Nicotiana clevelandii* leaves thus reducing virulence [7]. In another study the role of hemagglutinin genes from *Xylella fastidiosa*, the causal agent of several diseases in e.g. citrus, grape and peach was investigated. Random mutations of *X. fastidiosa* that produce hypervirulent strains with more severe symptoms and earlier grapevine death show an insertion in a hemagglutinin codifying gene. These results suggest that hemagglutinins mediate contact between bacterial cells impairing *X. fastidiosa* movement in the plant xylem and thus reducing virulence [8].

*Xanthomonas axonopodis* pv. *citri* is the phytopathogen that causes citrus canker. The disease appears as raised necrotic corky lesions in leaves, stems and fruits reducing fruit quality and quantity. The pathogen enters host plant tissues through stomata and wounds and infection is visualized as circular spots on the abaxial surface of leaves. Subsequently the bacteria colonize the apoplast causing the leaf epidermis to break due to cell hyperplasia [9], [10]. The complete bacterial genome has been sequenced [11] and reveals a number of genes most likely involved in virulence. Among them, we have previously characterized the role of the type III secretion system [12], the exopolysaccharide xanthan [13], and a plant natriuretic peptide-like molecule [14] in pathogenicity. *X. axonopodis* pv. *citri* has several types of protein secretion systems and besides a type I, two type II, one type III and one type IV secretion systems [15], [16], the phytopathogen bears at least one homolog to a TPS type V secretion system that may be involved in a FhaB-like hemagglutinin secretion. Here we characterize the function of this adhesin as well as its transporter partner in bacterial adherence, aggregation, motility, biofilm formation and virulence in the host plant.

## Results

### Sequence analyses of *XacFhaB* and other plant bacterial pathogens hemagglutinin-like genes

**Figure 1:**
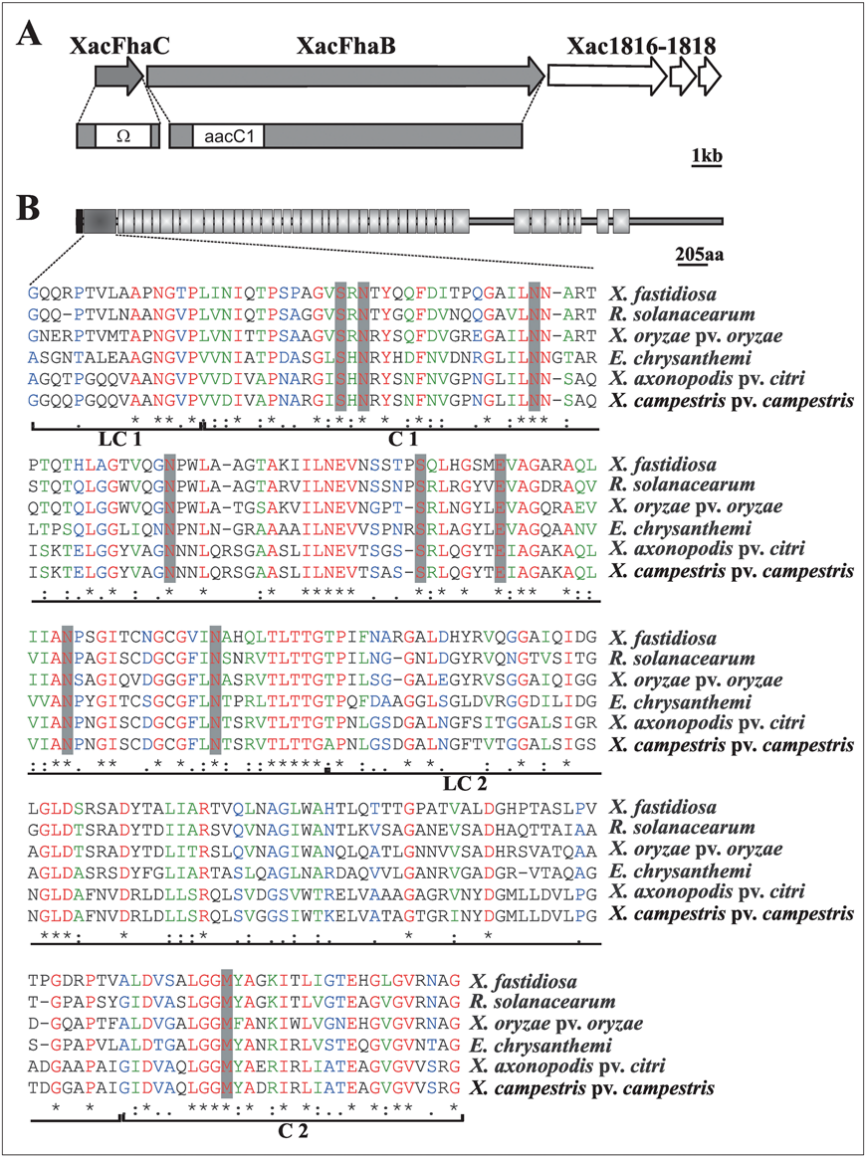
Schematic of the arrangement of *XacFhaB* genomic region and protein. (A) Representation of the genomic region containing *XacFhaB* gene; gray and white filled arrows correspond to open reading frames (ORFs). Also is shown the Δ*XacFhaC* and Δ*XacFhaB* constructions with the insertion of Sp/Sm and Gm cassettes respectively. (B) Representation of structural features of XacFhaB. Putative domains of the protein are depicted with black indicating the N-terminal signal peptide, gray indicating the conserved two-partner secretion domain and light gray indicating conserved repetitive domains. The two-partner secretion domains of FHA-like adhesins found in plant pathogens are aligned, and asterisks (*) signify identical amino acids, colons (:) are conservative replacements, full stops (.) are semi-conservative replacements. Residues that were found to be part of the secretion determinants are highlighted.

The *X. axonopodis* pv. *citri* hemagglutinin-like gene that we termed *XacFhaB* (XAC1815) encodes a predicted 4,753-aa protein. GenBank comparisons of XacFhaB revealed amino acid homology with putative adhesins of the hemagglutinin type represented by the FhaB from *B. pertussis* present in different plant and animal pathogens. Based on predicted amino acid homology and secondary structure, the gene found upstream of *XacFhaB* termed *XacFhaC* (XAC1814) encodes a putative TpsB secretion partner of XacFhaB protein (Figure 1A). Two other ORFs (Xac1816 and Xac1818) downstream of *XacFhaB* have been annotated in the *X. axonopodis* pv. *citri* genome sequence as hemagglutinin-like proteins [11] (Figure 1A). An alignment of these proteins and XacFhaB revealed that they encode disrupted proteins with an identity of 83% and 89% to the C-terminal end of XacFhaB. The absence of the N-terminal ends that include the TPS domain in Xac1816 and Xac1818 suggest that they are non-functional copies of the XacFhaB protein. Like other hemagglutinin proteins, XacFhaB has a signal peptide (predicted by SignalP: www.cbs.dtu.dk/services/SignalP/) that is followed by a conserved TPS domain. Comparison of XacFhaB TPS domain with hemagglutinin-like proteins present in other plant pathogens indicates that this domain is also conserved (Figure 1B). The TPS domain of XacFhaB is organized as typical TpsA proteins into four alternating regions, LC1–C1–LC2–C2 [4]. It is noteworthy that, even though XacFhaB and *B. pertussis* FhaB TPS domains are 36% identical, all residues that were found essential for secretion in the latter are also present in the XacFhaB TPS domain [17] (Figure 1B). Pattern and profile searches (http://pfam.sanger.ac.uk/search?tabsearchSequenceBlock) revealed that the XacFhaB TPS domain is followed by a number of conserved repeats predicted to form a repetitive β-strand structure, a feature shared with other TpsA proteins.

### Expression of *XacFhaB* and analyses of Δ*XacFhaB* and Δ*XacFhaC* mutant strains in citrus canker

**Figure 2:**
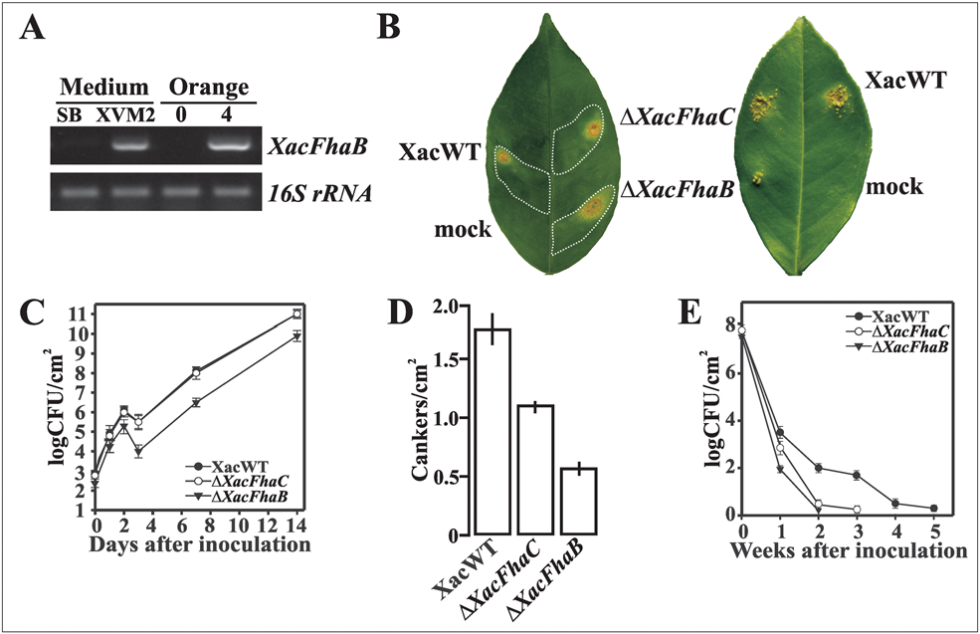
Expression of *XacFhaB* and effects of *XacFhaB* and *XacFhaC* mutations on pathogenicity. (A) RT-PCR of *XacFhaB* with RNA obtained from *X. axonopodis* pv. *citri* cultivated in SB or XVM2 medium (left gels) or recovered from inoculated orange at 0 or 4 days post-infection (right gels). As constitutive controls a fragment of 16S rRNA was amplified using the same RT-PCR conditions (bottom gels). (B) *X. axonopodis* pv. *citri* wild-type (XacWT) and knocked out strains Δ*XacFhaB* and Δ*XacFhaC*, were inoculated at 10^5^ cfu/ml (left leaf) or 10^4^ cfu/ml (right leaf) into the intercellular spaces of fully expanded orange leaves. A representative leaf is shown 7 days after inoculation. Dashed lines indicate the infiltrated area. (C) Bacterial growth of XacWT, Δ*XacFhaB* and Δ*XacFhaC* in orange leaves inoculated as described in A at 10^5^ cfu/ml, values represent means of three samples and are representative of three independent experiments. Error bars are standard deviations. (D) Quantification of canker number in orange leaves after one month of spray inoculation with XacWT, Δ*XacFhaB* and Δ*XacFhaC* strains at 10^9^ cfu/ml. Bars are the means of 20 leaves assayed and error bars are standard deviations, the results are representative of three independent experiments. (E) Epiphytic populations of XacWT, Δ*XacFhaB* and Δ*XacFhaC* strains on orange leaves. Each data point is the mean of four leaves assayed and error bars are standard deviations, the results are representative of five independent experiments.

To evaluate if *XacFhaB* is differentially expressed in the pathogenic process, we first analyzed its expression in rich and minimal medium by RT-PCR and observed expression induction only in XVM2 (Figure 2A), a nutrient poor medium that simulates conditions in the apoplastic space, corroborating previous results of macroarrays analysis in XVM2 medium [18]. We then evaluated if *XacFhaB* is expressed during plant-pathogen interaction. RNA was obtained from *X. axonopodis* pv. *citri* recovered from *Citrus sinensis* infected leaves. RT-PCR reactions showed that *XacFhaB* expression was hardly detected until 3 days post inoculation, slightly detected at day 3 (data not shown) and highly induced at 4 days after infiltration when symptoms start to appear in the infected leaves (Figure 2A) suggesting a role of XacFhaB in the pathogenicity process. As a control for constitutive bacterial expression a fragment of 16S rRNA was simultaneously amplified (Figure 2A). To ascertain the absence of plant RNA in bacterial samples controls with plant actin primers were carried out (data not shown).

In order to study the biological function of the hemagglutinin-like gene and its putative secretion partner, *XacFhaB* and *XacFhaC* deletion mutants were constructed from *X. axonopodis* pv. *citri* wild type strain by marker exchange mutagenesis (Figure 1A) and named Δ*XacFhaB* and Δ*XacFhaC*. The genomic arrangement of *XacFhaB* and *XacFhaC* points to a situation where downstream genes of *XacFhaB* have disrupted sequences probably preventing the making of functional proteins. Besides, upstream genes of *XacFhaC* are in opposite transcription orientation. As the mutants were generated by double recombination and each possesses a putative promoter sequence, it is unlikely that the phenotypes associated with the mutation of these genes are due to a polar effect. Accordingly, Δ*XacFhaC* has a different infection phenotype compared to Δ*XacFhaB* (see below). The virulence of these mutants was examined on the host plant *C. sinensis* using different inoculation methods. Upon pressure infiltration at a concentration ≥10^7^ cfu/ml, Δ*XacFhaB*, Δ*XacFhaC* and wild type bacteria induced similar disease symptoms and no differences were observed in the time of onset of lesion formation or lesion size (data not shown). However, when these bacteria were infiltrated at lower concentrations (10^5^ and 10^6^ cfu/ml) despite the fact that the infiltrated areas were similar, canker lesions (brown spots in Figure 2B) produced by Δ*XacFhaB* appeared in a more expanded manner than typical lesions observed with Δ*XacFhaC* or wild type bacteria (Figure 2B, left leaf). At lower bacterial concentration, 10^4^ cfu/ml, the virulence of Δ*XacFhaB* strain was strongly affected and produced a lesser number of canker lesions than the other strains (Figure 2B, right leaf).

To evaluate the role of XacFhaB in bacterial growth in the host, populations of *XacFhaB* and *XacFhaC* mutants and the wild type bacteria were quantified in orange leaves (Figure 2C). The results showed that at all times tested bacterial number of Δ*XacFhaB* recovered from the infected leaves was significantly lower than those of the wild type while no significant differences between the growth of the Δ*XacFhaC* and the wild type bacteria were observed (Figure 2C). The reduced bacterial number recovered from Δ*XacFhaB* infections was particularly larger after 3 days post-inoculation and may be explained by the fact that bacterial growth *in planta* requires *FhaB* expression and its absence may delay host colonization. Consistent with this, *XacFhaB* expression in wild type bacteria recovered from infected leaves was induced after this time and hardly detected before (see above).

The virulence of these strains was also determined after spraying them on leaves surfaces, a method that resembles the natural infection. After one month post inoculation the number of cankers on leaves infected with wild type bacteria was 3.2 and 1.7 times larger than that inoculated with Δ*XacFhaB* and Δ*XacFhaC* strains respectively (Figure 2D).

We also analyzed the epiphytic fitness of *XacFhaB* and *XacFhaC* deletion mutants compared to wild type bacteria and observed significant differences. As shown in Figure 2E, at 1 week from inoculation, the population size of wild type bacteria was nearly 40 and 5 times higher than both Δ*XacFhaB* and Δ*XacFhaC* populations. At 2 weeks, the differences in population were very pronounced and the wild type bacteria size was nearly 65 and 35 times higher than Δ*XacFhaB* and Δ*XacFhaC* populations (Figure 2E). At 2 weeks post inoculation Δ*XacFhaB* reached a minimum detectable population size whereas minimum values were observed after 3 weeks of inoculation with Δ*XacFhaC* and 5 weeks for wild type bacteria (Figure 2E). Collectively these findings suggest that XacFhaB has an important role in *X. axonopodis* pv. *citri* virulence and that this protein might be transported by XacFhaC, because of the intermediate infection phenotypes observed in Δ*XacFhaC* infiltrations we could speculate that another partner different from XacFhaC is involved in XacFhaB secretion.

### Influence of XacFhaB on cell attachment to different surfaces

**Figure 3:**
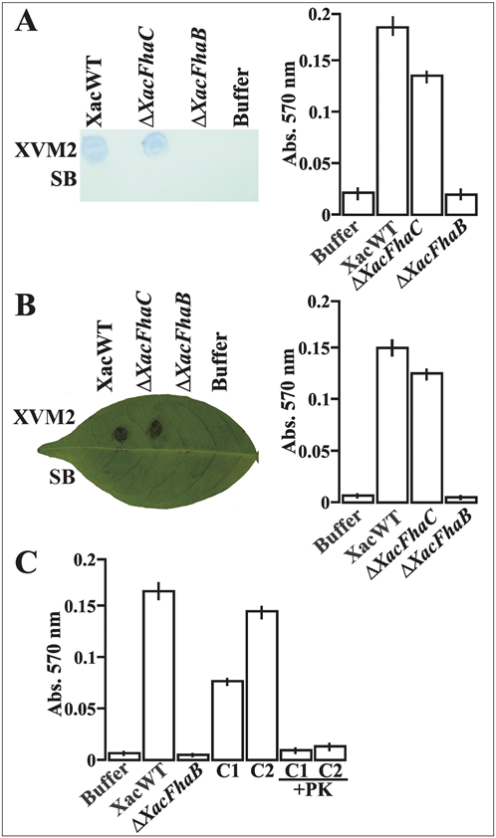
Adhesion of *X. axonopodis* pv. *citri* wild type and Δ*XacFhaB* and Δ*XacFhaC* strains. (A) Bacterial adhesion on plastic (PVC) surface of XacWT, Δ*XacFhaB* and Δ*XacFhaC* mutant strains grown in SB or XVM2 medium. In the left, a representative image of CV stain is shown. In the right, bars are the mean of CV stain measured spectrophotometrically (Abs. 570 nm) and error bars are standard deviations. The means were calculated from 20 wells (plastic) for each sample, the results are representative of three independent experiments. (B) Bacterial adhesion on abaxial leaves surfaces. In the left, a representative image of CV stain on a leaf is shown while in the right bars represent the mean of CV from 20 stained leaf spots measured as in (A) and error bars are standard deviations. (C) Adhesion of Δ*XacFhaB* to the leaf surface trough the co-incubation of this mutant strain with XacWT XVM2 supernatant (C1) or with XacWT cells grown in XVM2 in a relation 1∶1 (C2) without or with proteinase K (PK). Bars represent the mean of CV from 25 stained leaf spots measured as in (A) and error bars are standard deviations.

Having established that XacFhaB is necessary for *X. axonopodis* pv. *citri* virulence in citrus leaves, we next analyzed at which of the different stages of the pathogenic process XacFhaB might be involved. Initially, we investigated if XacFhaB enhances bacterial adherence and contact with plant tissue with an *in vitro* adhesion assay in 96-well polyvinylchloride (PVC) microtiter plates (Figure 3A). This assay was performed by incubating SB or XVM2 grown cultures of Δ*XacFhaB*, Δ*XacFhaC* and the wild type bacteria in the wells for six hours. Following the washing off of the planktonic cell fraction the remaining attached cells were stained with crystal violet (CV). Solubilization of the CV stain by addition of ethanol provides an indirect, quantitative measurement of the adherent cell mass in a given well [19]. The data obtained showed that the wild type strain adheres proficiently in XVM2 medium but not in SB medium (Figure 3A, left side). In SB medium the CV quantifications were similar to the negative buffer control (data not shown). In XVM2 medium Δ*XacFhaC* adhered slightly less well than the wild type, with a 1.5 fold lower levels of CV stain retained (Figure 3A, right side). In contrast, the Δ*XacFhaB* strain retained only basal levels of CV, similar to the negative buffer control (Figure 3A). A similar assay was performed on the abaxial surface of orange leaves (Figure 3B), and similar to what occurred on PVC surface, wild type and Δ*XacFhaC* strains attached to the leaves only if they were cultured in XVM2 medium, but Δ*XacFhaC* showed 1.5 fold lower CV retention. Furthermore, Δ*XacFhaB* again showed absence of adherence, indicating a general lack of adhesion (Figure 3B). As a consequence of the large size of *XacFhaB* gene we could not complement Δ*XacFhaB* deletion mutant, although we could partially revert the lack of adherence of this mutant in co-incubations with filtrated supernatants of wild type bacteria grown in XVM2 or with suspensions of the latter cultured in XVM2. Figure 3C shows that the adherence of Δ*XacFhaB* mutant to orange leaves co-incubated with the wild type bacterial supernatant reversed the lack of attachment of mutant cells (C1 in Figure 3C), suggesting that the XacFhaB protein secreted by the wild type strain contributed to the attachment of the mutant cells to orange leaves. Moreover, in the co-incubation of mutant and wild type bacteria previously washed to remove the respective culture medium (1∶1, being the total number of cells the same as in the XacWT) almost the same level of CV staining as with the wild type was observed, suggesting that mutant attachment to leaves was produced due to the presence of XacFhaB in wild type cells (C2 in Figure 3C). As a demonstration that this reversion was a result of the presence of an extracellular protein, the co-incubations C1 and C2 were treated with proteinase K (PK) before the inoculation of leaf surfaces. Under this regime PK prevented the attachment (Figure 3C). In all cases, analysis of the number of attached cells to leaf surfaces showed similar results to the CV stain (data not shown). All together these results suggest that XacFhaB, secreted or associated with bacterial membranes in a non-covalent manner, may mediate the attachment of *X. axonopodis* pv. *citri* to the leaf surface.

### The role of XacFhaB in biofilm formation

**Figure 4:**
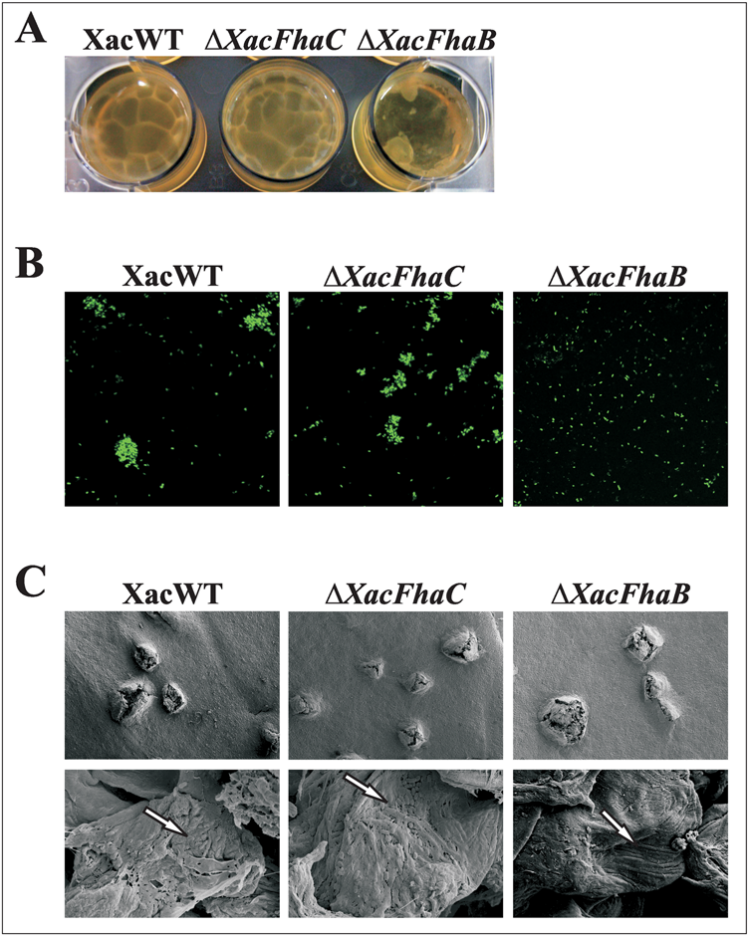
Biofilm formation of *X. axonopodis* pv. *citri* wild type and Δ*XacFhaB* and Δ*XacFhaC* strains. (A) Representative photograph of the biofilms formed by the *gfp*-expressing XacWT, Δ*XacFhaB* and Δ*XacFhaC* strains grown statically in 12-well PVC plates with SB medium. (B) Representative photographs of confocal laser scanning microscopy of the bacterial strains grown as in A. Magnification 100×. (C) Representative photographs of scanning electron microscopy of the different strains inoculated at a concentration of 10^5^ cfu/ml and grown for a period of 20 days in orange leaves. The upper panels shown the cankers formed by XacWT, Δ*XacFhaB* and Δ*XacFhaC* at 153× magnification, the lower panels correspond to a higher magnification (10000×). Arrows indicate the different arrays of cells.

We also questioned if the deficient adhesion phenotype of the *XacFhaB* mutant strain could also affect cell-to-cell aggregation and biofilm development by *X. axonopodis* pv. *citri*. Thus, we examined the *in vitro* biofilm characteristics of GFP-tagged wild type and Δ*XacFhaB* and Δ*XacFhaC* strains grown statically in 12-well PVC plates. After 14 days of growth the wild type bacteria and *XacFhaC* mutant were able to form structured biofilm in rich medium, in contrast the Δ*XacFhaB* showed an amorphous conformation (Figure 4A). Although bacteria were grown in rich medium in which we could not detected *XacFhaB* expression during the exponential growth phase, we might assume that after 2 weeks of growth at static incubation in the saturate culture where nutrients are limiting and population dynamics are changing, expression of *XacFhaB* may be taking place favoring biofilm assembly in the wild type bacteria. The bacteria present in the biofilm were transferred to glass slides and visualized by epifluorescence and confocal laser scanning microscopy. For the wild type and Δ*XacFhaC* strains, microcolonies were found scattered evenly throughout the field of view but absent in Δ*XacFhaB* (Figure 4B). Similar results were observed when these bacteria were grown in XVM2 medium (data not shown). The different behaviors of wild type, Δ*XacFhaB* and Δ*XacFhaC* cells *in planta* were observed by scanning electron microscopy. The cankers observed on the abaxial leaf surface at 153× magnification of the wild type and Δ*XacFhaC* bacteria were different to those observed for the Δ*XacFhaB* mutant. The latter displayed less compact and more cavities inside the canker structure (Figure 4C, upper panels). When we observed the infected tissues at higher magnifications (10000×), the wild type and Δ*XacFhaC* formed clusters of aggregated cells that were arranged in a similar manner as in biofilms of *Xylella fastidiosa* during infection of grapevine vessels (Figure 4C, lower panels) [8]. The *XacFhaB* mutant cells did not appear to aggregate in such a manner and showed a lack of three-dimensional array in the apoplastic space of leaves compared to the wild type (Figure 4C). These findings suggest that *XacFhaB* is also necessary for cell-to-cell attachment mediating *in vitro* and *in vivo* biofilm formation by *X. axonopodis* pv. *citri*.

### The role of XacFhaB in cell motility

**Figure 5:**
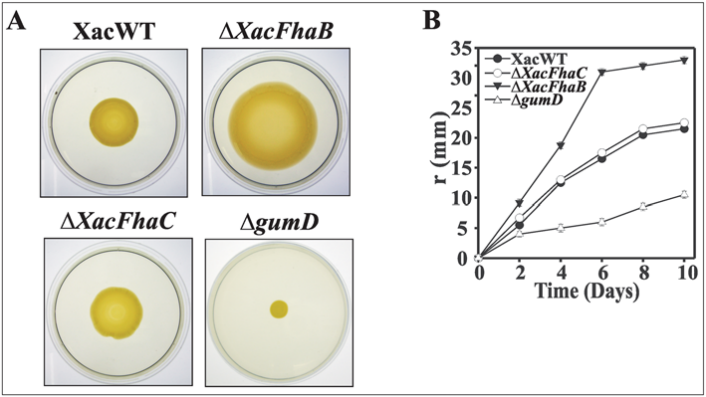
Swarming motility of *X. axonopodis* pv. *citri* wild type and Δ*XacFhaB*, Δ*XacFhaC* and Δ*gumD* strains. (A) The different strains were centrally inoculated on SB plates fortified with 0.7% agar. After 6 days incubation at 28°C the plates were photographed on a light transiluminator to denote the different swarming phenotype. (B) The motility zone of swarming colonies grown as in A was measured at different times of incubation during a period of 10 days. Each data point showed in the figure is an average of 4 independent experiments, error bars indicate the standard error. The results are representative of four independent experiments.

Since canker lesions formed by Δ*XacFhaB* mutant occurred at further distances from the point of infiltration compared to lesions caused by the wild type (Figure 2B, left leaf), we evaluated if XacFhaB could affect cell motility by swarm plate analysis (Figure 5). This analysis showed that cells of the wild type and Δ*XacFhaB* and Δ*XacFhaC* strains were motile on semi-solid swarm SB medium (Figure 5A). The motility zone of the wild-type and mutant cells was measured at different times of incubation (Figure 5B). For all the strains the movement was slow on the first day of culture, but got faster after 2 days (Figure 5B). This type of movement suggests that motility of *X. axonopodis* pv. *citri* is inducible, a phenomenon that has been seen in typical swarming motility of other bacteria. At all times recorded the Δ*XacFhaB* moved faster and further than wild type and Δ*XacFhaC* strains. Bacterial phenotypes were also different in the strains tested, showing higher layers of cells in wild type and Δ*XacFhaC* compared to Δ*XacFhaB* (Figure 5A). Even though this assay was performed in rich medium, differences in motility were observed after 2 days in semi-solid culture and we suggest that under these conditions XacFhaB is being expressed in wild type bacteria restraining its movement. In other bacteria swarming involves differentiation into an elongated cell type, hyperflagellation and the production of slime. We analyzed the swarming capacity of a strain mutated in *gumD*, the gene that encodes the first protein involved in the biosynthesis of the extracellular exopolysaccharide (EPS), that could not produce xanthan [13]. Δ*gumD* was impaired in swarming motility, demonstrating that *X. axonopodis* pv. *citri* motility depends on the EPS that may function as a friction-reducing molecule (Figure 5A and B). Moreover, when swarming motility was analyzed on XVM2 semi-solid plates, wild type and Δ*XacFhaC* did not move probably due to the absence of EPS production in this medium (data not shown), while Δ*XacFhaB* advanced a slight distance from the inoculation point suggesting that regardless of the presence of EPS XacFhaB could have an noticeable effect on cell motility under these conditions.

**Figure 6:**
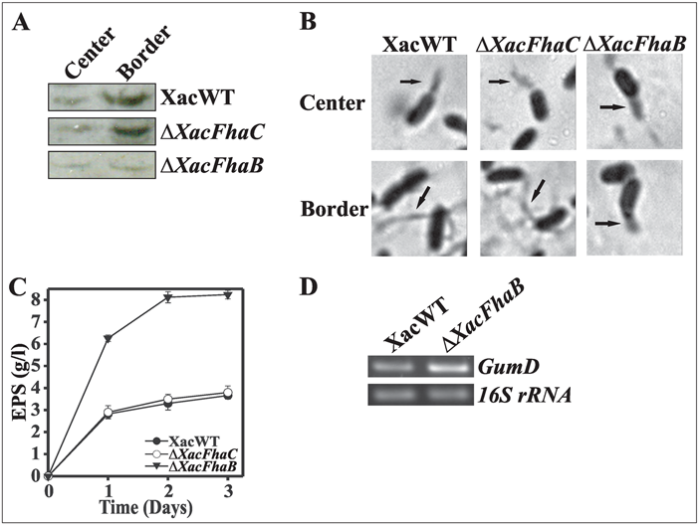
Analysis of the factors involved in the swarming motility of *X. axonopodis* pv. *citri* wild type and Δ*XacFhaB* and Δ*XacFhaC* strains. (A) For flagellar proteins immunodetection, whole-cell extracts from the XacWT, Δ*XacFhaB* and Δ*XacFhaC* strains isolated from the center (C) or the border (B) of the swarming colony grown as described in Figure 5A were analyzed by Western blotting developed with anti-flagellin polyclonal antibodies. Samples were standardized as described in Materials and Methods. (B) Cells of XacWT, Δ*XacFhaB* and Δ*XacFhaC* strains isolated from the center or the border of the advancing swarm were stained for flagellar structures (indicated by arrows) and observed under light microscopy at 100× magnification. The photographs are representative of three experiments in which several fields of view were observed. (C) Xanthan production in XOL medium of XacWT, Δ*XacFhaB* and Δ*XacFhaC* mutants strains. Each data point is the mean of three experiments, error bars indicate the standard error. (D) Total RNA was extracted from XacWT and Δ*XacFhaB* mutant grown in SB for 48 h at 28°C and *gumD* expression was analyzed by RT-PCR using specific primers. 16S rRNA was used as a constitutive control.

To determine whether flagellum biosynthesis is required for swarming in *X. axonopodis* pv. *citri*, the expression of flagellar proteins was analyzed by Western blot assay (Figure 6A) [20]. Expression of these proteins in wild type bacteria increased from the swarm center to the border, suggesting that hyperflagellation drives swarming motility. Similar increases in flagellar proteins were observed in Δ*XacFhaC* cells which moved like wild type bacteria. Nevertheless, in the *XacFhaB* mutant that showed faster movements in swarm plates, flagellar protein expression was lower as compared to the wild type and *XacFhaC* mutant strains (Figure 6A). We also analyzed the flagellar structures present in the wild type and mutant bacteria (Figure 6B). In all cases the stain of flagellar structures revealed the presence of single flagellum in the cells obtained from the swarm center (Figure 6A, upper panels). As observed in other bacteria [21], wild type and Δ*XacFhaC* bacteria from the swarm border were more elongated and hyperflagellated. Conversely, Δ*XacFhaB* bacteria from the swarm border retained the phenotype of the swarm center (Figure 6A, lower panels). Since Δ*XacFhaB* did not show a hyperflagellated phenotype we questioned if the differential motility was the consequence of the presence of increased slime secretion in the mutant. To test this hypothesis EPS production was measured in wild-type, Δ*XacFhaC* and Δ*XacFhaB* strains in XOL medium supplemented with 1% glucose, an optimal medium for xanthan quantification [13]. Maximal values for the latter were of 8.2 g/l while for wild type and Δ*XacFhaC* strains were of ∼3.8 g/l (Figure 6C). The expression of the *gumD* gene was assayed by RT-PCR. RNA used for the RT-PCR reactions was obtained from wild type and Δ*XacFhaB* strains cultured for 48 h in SB medium. In the *XacFhaB* mutant *gumD* transcript levels were close to 90% higher than in the wild type (Figure 6D), being consistent with the observed increase in EPS production (Figure 6C). As a control for constitutive bacterial expression a fragment of 16S rRNA was simultaneously amplified (Figure 6D). Similar results were obtained for bacteria grown on XOL, while no *gumD* expression was observed in any strain grown in XVM2 (data not shown). Taken together, these results clearly show that the increased motility phenotype observed in the *XacFhaB* mutant strain is due to a lack of adhesion together with the larger amount of EPS present, and not an increase in flagellar proteins.

## Discussion

Bacterial pathogen adhesion to animal tissues is a key step in the colonization of the host tissue and particularly the involvement of adhesins in these interactions has been studied extensively in recent years. In contrast the role of plant bacterial pathogen adhesins in pathogenicity has been only characterized for a few plant pathogen interactions [7], [8], [22]. In this study we analyzed the role of FhaB and FhaC, components of the two partner secretion system in the interaction between *X. axonopodis* pv. *citri* and orange, its host plant. *FhaB* expression was observed in bacteria grown in apoplastic space mimicking XVM2 medium and also when the bacteria were recovered from infected leaves, suggesting a role in pathogenicity. Consistent with this, promoter analysis of *FhaB* previously revealed the presence of an imperfect PIP box, a conserved plant-inducible promoter sequence motif [23] and an 8-bp conserved motif that is present in the promoter region of all genes induced in XVM2 medium [18]. The *FhaB* deletion mutant was affected in virulence, both in epiphytic and wound inoculations. The mutant infection displayed more dispersed lesions and a lesser number of cankers. Moreover, the number of bacterial cells recovered from the leaf apoplast was less than in the wild type infection and epiphytic survival was also impaired in the Δ*XacFhaB* strain. These results are consistent with less bacterial adhesion found on the leaf surface as well as on plastic material (PVC). This indicates that XacFhaB has an important function in *X. axonopodis* pv. *citri* virulence at the early stage of pathogenesis when the bacteria has to attach to the leaf surface and later when the bacteria has to survive inside the plant tissue. Our results are in agreement with those previously reported for *E. chrysanthemi*, in which mutants in *hecA*, the hemagglutinin homolog to *XacFhaB*, had impaired ability to attach to leaves and form aggregates on them and cause an aggregate-associated killing of epidermal cells [7]. Our results answer the previously open question if hemagglutinin-like proteins from a phytopathogen are important when inoculum is applied to the surface of a susceptible host rather than to a wound [7]. Here we demonstrate that *FhaB* deletion mutant inoculation into the host leaf tissue produces less severe symptoms, indicating that adhesin proteins are not only involved in bacterial attachment to the host surface but also in causing the virulence phenotype in the plant tissue.

Based on predicted amino acid homology, secondary structures and vicinity to *XacFhaB*, we hypothesized that the gene *XacFhaC* may be the transporter protein of XacFhaB. However, the *XacFhaC* mutant showed an intermediate phenotype in spray inoculation, epiphytic fitness and adherence to PVC plates and orange leaves, while resembling the wild type strain in infiltration inoculations and growth inside the leaf, biofilm formation, swarming motility and hyperflagellation phenotype. These differences may be attributed to the fact that XacFhaB could be secreted by another partner different from XacFhaC. In human and animal pathogens TpsB transporters appear to be specific for their respective TpsA partners and the encoding genes localized adjacent to each other [24]. Our results indicate that in plant pathogens this may not be the case. Accordingly, in *X. fastidiosa* it was proposed that only one putative *TpsB* transporter protein encoding gene is distantly localized from the *HxfA* and *HxfB* adhesin genes [8]. By searching the non-redundant database for similar proteins using BLAST, other *X. axonopodis* pv. *citri* proteins with homology to TpsB proteins were found. An example is the protein encoded by the XAC4114 gene that was classified as hemolysin activator protein (ShlB). Although XAC4114 is located distantly in the chromosome from *XacFhaB*, we may suggest that it might also mediate the transport of XacFhaB through the external membrane, a hypothesis that will be tested in the future. Our results showed that the *XacFhaC* mutant was impaired in plant tissue attachment and epiphytic fitness while no differences were observed in bacterial cell-to-cell attachment, suggesting that the amount of XacFhaB possibly secreted by other transporter proteins may be sufficient for the interaction between bacterial cells while higher levels are required to interact with the plant surface.

Bacteria use a variety of motility mechanisms to colonize environments, including flagella-dependent swimming and swarming, and flagella-independent, twitching, gliding, and sliding [25]. Previous studies have shown that other Xanthomonas strains have swarming motility in semi-solid agar [26], [27], but little is known about the mechanism implicated in this process. Here we provide evidence that *X. axonopodis* pv. *citri* swarming cells have a distinct morphology, being generally longer and more flagellated than vegetative cells, and that swarming motility depends on EPS secretion to the medium. Our results suggest that XacFhaB may have an effect on swarming motility since bacteria that do not have this adhesin display a different swarming phenotype and move faster than wild type bacteria. Nevertheless, Δ*XacFhaB* has fewer flagellar surface filaments than wild type *X. axonopodis* pv. *citri* as determined by filament protein levels and flagella staining. These results are consistent with the idea that increased bacterial movement is due to the lack of aggregation between cells that in turn remits faster advance. In addition, we observed that *XacFhaB* mutant produced higher levels of EPS than wild type bacteria and speculate that the net decrease in cell-to-cell interaction due to the absence of XacFhaB adhesin and higher levels of EPS secreted facilitate bacterial translocation. Several bacteria can rapidly colonize the surface of semi-solid media in a flagellum-independent manner while surface colonization depends on the secretion of surfactant agents. Such a movement is known as “sliding motility” and has been studied extensively in *Mycobacterium smegmatis* [28], *Bacillus subtilis* [29] and *Pseudomonas aeruginosa* [30]. Since the absence of XacFhaB and higher EPS levels may favour bacterial motility we suggest that *XacFhaB* mutant may not need to be hyperflagellated. Studies in *Salmonella typhimurium* on swarm agar plates show a down-regulation of flagellar genes activated by a feedback mechanism in which the flagellar filament itself is a sensor for unfavorable external conditions [31].

Several of the studies performed in this work, in particular biofilm formation, swarming motility, flagella and EPS production, and *gumD* expression were analyzed in bacteria grown in rich medium. In the case of biofilm formation we observed the same lack of aggregation for Δ*XacFhaB* cells in experiments performed in SB or XVM2. In swarming motility assays, differences among strains were observed in rich medium while in minimal medium only Δ*XacFhaB* advanced slightly. Examination of flagella was performed on cells obtained from SB plates in which the motility could be quantified. By contrast neither EPS production nor *gumD* expression could be observed in bacteria grown in XVM2. The absence of *gumD* expression in XVM2 could account for the less structured biofilm and for the lack of movement observed for the wild type bacteria in this medium. How could these results be obtained in rich medium in which FhaB has an active role if *FhaB* expression can not be detected in bacteria grown in it? We propose that the lack of *FhaB* expression in liquid SB cultures at exponential phase may not be representative for all growth condition in this medium and in the case of biofilm formation we suggest that after 2 weeks of growth at static incubation, bacteria reach a population density that may trigger the expression of the gene. Similarly, in the semi-solid agar plates, adhesin expression in wild type cells could be taking place as bacteria have to remain attached to each other in this type of growth.

Bacterial cell-to-cell aggregation may be critical for the interaction with the plant. In this particular plant-pathogen interaction in which the bacteria causes a local disease, *X. axonopodis* pv. *citri FhaB* mutant showed more expanded lesions suggesting that XacFhaB is required to confine the bacteria within a small radius near the initial site of infection. The lack of adhesion between cells was also observed in *X. fastidiosa* mutants in hemagglutinin genes, but in this latter case the phytopathogen showed increased virulence. This may be attributed to the fact that *X. fastidiosa* colonizes grape xylem vessel resulting in a systemic disease, and thus the lack of hemagglutinin proteins may well favor bacterial movement and colonization of vascular tissue [8]. A more recent report suggests that movement and multiplication of *X. fastidiosa* in plants are linked, perhaps because cell wall degradation products are the major source of nutrients and cell-to-cell interaction which restricts movement and colonization of *X. fastidiosa* may be an adaptation to endophytic growth of the pathogen that prevents the excessive growth of cells in vessels [32].

Biofilms are matrix-enclosed bacterial populations that adhere to each other, surfaces or interfaces. A key aspect of biofilm formation is the production of the extracellular matrix. In bacteria like Pseudomonas, this matrix is thought to be comprised of EPS, DNA, and proteins [33]. The main proteins identified in this matrix were alpha-hemolysin [34] and adhesin proteins [35], [36]. Accordingly, in a microarray analysis of genes being expressed in *X. fastidiosa* cells growing in a biofilm compared to planktonic cells, de Souza et al. identified genes that encode hemagglutinin-like secreted proteins, supporting the idea that proteins involved in adhesion to surfaces are implicated in biofilm formation [37]. We observed that *X. axonopodis* pv. *citri* mutant in hemagglutinin-like protein, that overproduces xanthan, hindered biofilm formation suggesting that EPS can not be the only one responsible for biofilm formation and that XacFhaB adhesin is also essential for this process. The higher amount of xanthan produced by this mutant may be explained by a mechanism of compensation for the lack of adherence, though it is not enough by itself to promote the formation of the biofilm, suggesting that the xanthan required for biofilm formation may be maintained in the extracellular matrix in an adhesin-dependent manner. Biofilm formation and swarming motility are both surface-associated types of behavior and bacteria must be capable of alternating from one behavior to the other. ÓToole and collaborators have established the mechanisms that co-regulate and inversely control swarming and biofilm formation in *Pseudomonas aeruginosa* [38]–[40]. These authors proposed that the proteins involved in such regulation are SadC and BifA, a diguanylate cyclase and cyclic-di-GMP phosphodiesterase, respectively and that they function together to modulate the levels of c-di-GMP in the cell. The resulting levels of this molecule impact on both EPS production and flagellar function and thus biofilm formation and swarming motility. Increased levels of c-di-GMP are usually correlated with increased exopolysaccharide production and increased biofilm formation, whereas lower levels of c-di-GMP are seen to increase motility. The same group also demonstrated that c-di-GMP levels that are lowered in a phosphate limiting environment, regulate secretion of the adhesin LapA required for biofilm formation by *Pseudomonas fluorescens* and further extended the biological roles of c-di-GMP beyond regulation of exopolysaccharide production and motility [41]. Our results suggest that in *X. axonopodis* pv. *citri* the action of XacFhaB adhesin is causing reduced bacterial migration favouring biofilm formation, and since several proteins involved in regulating c-di-GMP levels are codified in the citrus pathogen genome we may speculate that they could also coordinate adhesin and EPS mediated attachment, modulating *X. axonopodis* pv. *citri* biofilm formation and swarming motility and thus its lifestyle. Another regulatory mechanism proposed by Lindow and collaborators implies that adhesin proteins expression could be regulated by the two-component signaling system Rpf that also coordinates the expression of virulence genes such as the ones involved in EPS production [32]. The higher amount of EPS observed in *XacFhaB* mutants may be attributed to the adaptation of this regulatory pathway. Our future aims are to elucidate the pathways that may coordinate and regulate the action of these extracellular macromolecules of *X. axonopodis* pv. *citri* in citrus canker.

## Materials and Methods

### Bacterial Strains, Culture Conditions and Media

*Escherichia coli* cells were cultivated at 37°C in Luria Bertani (LB) medium. *X. axonopodis* pv. *citri* and mutant strains were grown at 28°C in Silva Buddenhagen (SB) medium (5 g/l sucrose, 5 g/l yeast extract, 5 g/l peptone, and 1 g/l glutamic acid, pH 7.0), XVM2 medium (20 mM NaCl, 10 mM (NH_4_)_2_SO_4_, 1 mM CaCl_2_, 0.01 mM FeSO_4_, 5 mM MgSO_4_, 0.16 mM KH_2_PO_4_, 0.32 mM K_2_HPO_4_, 10 mM fructose, 10 mM sucrose and 0.03% casein acid hydrolysate (casaminoacid), pH 6.7), or XOL (K_2_HPO_4_ 4 mM, KH_2_PO_4_ 1.5 mM, (NH_4_)_2_SO_4_ 1 g/l, FeSO_4_ 0.01 g/l, MnCl_2_ 5 mM, MgCl_2_ 0.5 mM, tryptone 1.25 g/l, yeast extract 1.25 g/l, and glucose 1 g/l. Antibiotics were used at the following final concentrations: ampicillin, (Ap) 100 µg/ml for *E. coli* and 25 µg/ml for *X. axonopodis* pv. *citri*, gentamicin, (Gm) 20 µg/ml for *E. coli* and 4 µg/ml for *X. axonopodis* pv. *citri*, streptomycin (Sm), 100 µg/ml for *E. coli* and 50 µg/ml for *X. axonopodis* pv. *citri*, spectinomycin (Sp), 50 µg/ml for *E. coli* and 25 µg/ml for *X. axonopodis* pv. *citri*, (Km) 40 µg/ml for *E. coli* and 25 µg/ml for *X. axonopodis* pv. *citri*. *X. axonopodis* pv. *citri* strain Xcc99-1330 was kindly provided by Blanca I. Canteros (INTA Bella Vista, Argentina).

### Recombinant DNA and Microbiological Techniques

All DNA manipulations including the alkaline method of plasmid purification, restriction enzyme digestion, ligation and gel electrophoresis were performed with standard techniques [42], unless otherwise specified. Total bacterial genomic DNA from *X. axonopodis* pv. *citri* was isolated using the cetyltrimethylammonium bromide procedure [43]. For bacterial conjugations plasmids were transferred to *X. axonopodis* pv. *citri* by biparental mating from the broad host-range-mobilizing *E. coli* strain S17-1. Bacterial mixtures were spotted onto Hybond-C membranes, placed on Nutrient agar and incubated for 48 h at 28°C. The membranes were then washed and the bacteria transferred to selective medium as previously described [12].

### Generation of *XacFhaB* and *XacFhaC* Deletion Mutants

The flanking regions of Xac1814 and Xac1815, codifying *XacFhaC* and *XacFhaB* respectively, were amplified by PCR using two pairs of oligonucleotides. The pair used for the amplification of the region upstream Xac1814 were 5′XAC1814up (5′ ACGCGTCGACGTGAGTGCTGCAATGCTTGAC 3′) and 5′XAC1814down (5′ ATACAAGCTTGATCGCATCCACATCTGTCCT 3′) rendering a fragment of 1296 bp. Underlined are the restriction sites for *Sal*I and *Hind*III, respectively. The pair used for the amplification of the region downstream Xac1814 was 3′XAC1814up (5′ ATCAGAATTCGTACTGGCGCAATACCTTCAG 3′) and 3′XAC1814down (5′ ATCAGGATCCGTTGGTTCCACTCAGGCTCAAT 3′) rendering a fragment of 1238 bp. Underlined are the restriction sites for *Eco*RI and *Bam*HI, respectively. This fragment was used as the upstream region of Xac1815, the pair used for the amplification of the region downstream Xac1815 was 3′XAC1815up (5′ ACGCGTCGACAGGGCGATATGACCTTGCAG 3′) and 3′XAC1815down (5′ ATACAAGCTTCATCGATGCTCAGCTGGTTAG 3′) rendering a fragment of 1410 bp. Underlined are the restriction sites for *Sal*I and *Hind*III, respectively. Genomic DNA (100 ng) was used as the template in PCR (50 µl reaction volume) performed in an Eppendorf thermal cycler, with denaturation at 94°C for 3 min, followed by 30 cycles of 94°C for 1 min, 58°C for 1 min, and 72°C for 2 min, and final extension at 72°C for 10 min. Amplified products of Xac1814 were cloned sequentially in pBluescript II KS+ (Stratagene) previously digested with *Sal*I and *Hind*III and then with *Eco*RI and *Bam*HI. The resulting plasmid with both Xac1814 flanking regions was digested with *Hind*III and in this site the 2 kbp Ω cassette coding for Sm/Sp resistance from pKRP13 [44] was subcloned. The recombinant plasmid pBS5Ω3Xac1814 was digested with *Sal*I and *Bam*HI and cloned in pK19mobGII [45], rendering pK195Ω3Xac1814. Amplified products of Xac1815 were cloned sequentially in pK19mobGII previously digested with *Eco*RI and *Bam*HI and then with *Sal*I and *Hind*III. The resulting plasmid with both Xac1815 flanking regions was digested with *Bam*HI and in this site the 2.3 kbp aacC1 cassette coding for Gm resistance was subcloned rendering pK195(aacC1)3Xac1815. *E. coli* S17-1 cells transformed with pK195Ω3Xac1814 and pK195(aacC1)3Xac1815 were conjugated to *X. axonopodis* pv. *citri* and selected for Sp/Sm and Gm resistance and Km sensitivity to obtain Δ*XacFhaC* and Δ*XacFhaB* deletion mutants, respectively.

### RNA Preparation and RT-PCR

For the analysis of *XacFhaB* expression total RNA of *X. axonopodis* pv. *citri* cells cultured in either SB or XVM2 medium at exponential phase was isolated using TRIzol® reagent (Invitrogen) according to the manufacturer's instructions. RNA preparations of bacteria from inoculated leaves at 0 and 4 days post infection was done as described previously [46]. Briefly, 20 leaves of orange were inoculated with *X. axonopodis* pv. *citri* and 10 leaves at each time of infection were collected, decontaminated with alcohol, and the midribs were removed. The leaves were cut into pieces with a sterile razor blade and maintained for 1 h in sterile glass plates containing 20 ml of distilled water to allow the bacteria to exudate from the leaf tissue to the water. The leaves were separated from the suspension by pipetting the water, which was centrifuged to pellet the bacterial cells. The bacteria were washed with water and used for RNA isolation using TRIzol® reagent (Invitrogen). After treatment with DNAse (Promega), cDNA was synthesized from 1 µg of total RNA using MMLV RT (Promega) and the oligonucleotide dN6. To analyze the expression of *XacFhaB*, PCR was done with 1 µl cDNA template using the pair of oligonucleotides: 3′XAC1815up and 3′XAC1815down under the following conditions: 94°C for 2 min, followed by 30 cycles of 94°C for 1 min, 58°C for 1 min, and 72°C for 1.5 min, and final extension at 72°C for 10 min. As a constitutive control a 217 bp fragment of 16S rRNA was amplified using the same PCR conditions with the pair of oligonucleotides: 16S rRNA up (5′ TGGTAGTCCACGCCCTAAACG 3′) and 16S rRNA down (5′ CTGGAAAGTTCCGTGGATGTC 3′). To detect any plant RNA contamination the pair of oligonucleotides: ActinL (5′ ACGTGAATTCTAGTGTTTCGATAAGT 3′) and ActinR (5′ TCAATTGGATACTTCAAAGTCAAAAT 3′) that amplified a fragment of 800 bp of the plant actin gene were used in a similar PCR reaction. For the analysis of *XacGumD* expression RNA was obtained from *X. axonopodis* pv. *citri* and Δ*XacFhaB* cells cultured in SB medium during 48 h. RT-PCR was done using the pair of oligonucleotides: GumDup (5′ GCGCGGCCGTGGGATTGCTGAGT 3′) and GumDdown (5′ TGGCGGCGCTGACGGAAGAACAC 3′) under the PCR conditions described previously. As a constitutive control the same fragment of 16S rRNA was amplified.

### Plant Material and Inoculations

Orange (*Citrus sinensis* cv. Valencia) was used as the host plant for *X. axonopodis* pv. *citri*. All plants were grown in a growth chamber in incandescent light at 28°C with a photoperiod of 16 h. Bacteria were cultured in SB broth to an optical density at 600 nm (OD_600_) of 1, harvested by centrifugation, and resuspended in 10 mM MgCl_2_ at 10^4^ to 10^7^ cfu/ml. For disease symptoms assays, bacterial suspensions were infiltrated into leaves with needleless syringes. Also, bacteria inoculations were made by spraying on orange leaves. *In planta* growth assays were performed by grinding 0.8 cm diameter leaf discs from infiltrated leaves in 1 ml of 10 mM MgCl_2_, followed by serial dilutions, and plating onto SB agar plates. Colonies were counted after 48 h of incubation at 28°C, and the results are presented as cfu per cm^2^ of leaf tissue. Cankers were count from 20 orange leaves inoculated with the different strains and the areas of the counted leaves were measured from digitalized images using Adobe Photoshop software. Epiphytic fitness was evaluated as previously described [13]. Briefly, bacteria were sprayed onto leaves at a concentration of 10^7^ cfu/ml in 10 mM potassium phosphate buffer (pH 7.0), until both leaf surfaces were uniformly wet. Three leaf samples (1.5 cm2 per time-point) were taken at different days after inoculation and transferred to 0.4 ml of 10 mM potassium phosphate buffer (pH 7.0) in 1.5 ml Eppendorf tubes. Tubes were submerged in a Branson model #5510 sonicator for 10 min. Subsequently, each tube was vortexed for 5 s, and serial dilutions plated on SB plates containing Ap to count *X. axonopodis* pv. *citri* wild type and Δ*XacFhaB* and Δ*XacFhaC* colonies. In all cases, each data point showed in the figures is an average of 3 independent experiments. Error bars indicate the standard deviations (p<0.01).

### Adhesion Assays

To measure the level of cells adhered to a plastic surface, overnight cultures in SB or XVM2 medium of the different strains were centrifuged to recovered cell pellets, washed and resuspended in phosphate buffer (pH 7.0) to the same OD_600_. Then, 200 µl of each bacterial suspension were aliquot to each well of 96-well polyvinylchloride (PVC) including 20 wells with buffer (negative control) and incubated for 6 h at 28°C. The adherence was monitored by staining with crystal violet (CV) [47]. Bacterial adhesion was measured after repetitive washing of the plates to removed non-adherent cells and staining with 1% CV for 15 min at room temperature. Excess stain was removed by washing under running tap water, and the CV stain was solubilized by the addition of 150 µl volumes of 95% ethanol added to each well. CV was quantified with a microplate reader at 570 nm absorption wavelength. To analyzed bacterial adherence to leaves surface 20 µl of each bacteria suspension were incubated for 6 h at 28°C in a humidified chamber on the abaxial face. Bacterial adhesion was measured by CV stain as for the plastic surface. For complementation assay wild type bacteria grown in XVM2 were centrifuged and the resulting supernatants were filtrated trough a 0.45 µm filter unit (Minisart®) to eliminate remaining bacterial cells. Δ*XacFhaB* cells grown in XVM2 were centrifuged and resuspended in the wild type supernatant. The adhesion of Δ*XacFhaB* cells in these suspensions was analyzed as described before. The adherence of Δ*XacFhaB* cells was also assayed in the presence of wild type bacteria, to this end, bacteria were cultured in XVM2, centrifuged, washed to remove the culture medium and resuspended in PBS in a relation of 1∶1, being the total number of cells the same as the number of XacWT cells used as a positive control of adhesion. In these co-incubation experiments proteinase K was added to the *X. axonopodis* pv. *citri* wild type suspensions or to the XVM2 supernatants at a final concentration of 100 µg/µl and incubated for 30 min at room temperature before the adherence assay. In all cases, each data point is an average of 20 wells or 20 spots on leaves surface, and error bars indicate the standard error (p<0.05). Representative data from one of at least three independent experiments are shown.

### *In vitro* Biofilm Assays

For the analysis of biofilm formation all the strains were modified to express the green fluorescence protein (GFP). To this end, the coding sequence for EGFP from pEGFP-1 (Clontech, Palo Alto, CA, U.S.A) was digested with *BamH*I and *Xba*I and ligated in frame with the LacZ-α-peptide of the broad-host-range vector pBBR1MCS-2 [48] previously digested with the same enzymes, rendering the plasmid pBBR1MCS-2EGFP. *E. coli* S17-1 cells transformed with this plasmid were conjugated to the different strains and selected for Km resistance. The obtained *gfp*-expressing bacterial strains were grown in SB medium to the stationary phase. Then, 20 µl of each culture was diluted in 2 ml of fresh SB or XVM2 medium and loaded on 12-well PVC plates. All plates were statically incubated at 28°C for 14 days, then samples of bacterial from the formed biofilms were transferred to glass slides and visualized by confocal laser scanning microscopy (Nikon Eclipse TE-2000-E2).

### *In vivo* Biofilm Assays

In order to analyzed biofilm formation in plant, scanning electron microscopy was used. Orange leaves infected with the different strains at a concentration of 10^5^ cfu/ml for a period of 20 day were cross-sectioned to 1-mm thickness on a dental wax pad. Sections were fixed in 2.5% glutaraldehyde in 0.1 M phosphate buffer, pH 7.2. In the Electron Microscopy Lab the fixed sections were subjected to dehydration by increasing the amounts of ethanol (20%, 50%, 75%, 15 s each step and three times: 100%, 30 min, after that 30 s in acetone 50% and 100%). After 100% acetone treatment the tissue was critical point dried in a Polaron apparatus. Samples were mounted in aluminium stubs with double stick tape and coated with gold in a sputter coater Pelco 9100. The observation was made in a Scanning Electron Microscope EVO 40 (Leo, Cambridge, UK) at 5 KV. Scanning electron microscopy analysis was performed twice, with three replicates for each sample.

### Swarming Assays

For swarming motility, SB plates fortified with 0.7% agar and dried during 1 h were inoculated with 2 µl of 10^7^ cells/ml grown to mid-log phase at 28°C in SB broth. The inoculated Petri dishes were then incubated at 28°C for 10 days and the motility zone was measured at different times of incubation. Each data point showed in the figure is an average of 4 independent experiments. Bar errors indicate the standard error (p<0.05).

### Flagellar proteins analysis

Flagellar proteins levels were determined by Western blot analysis using anti-flagellin rabbit polyclonal antibodies [20]. For the swimming assays, bacterial cultures grown overnight in SB at 28°C were centrifuged and resuspended in PBS at the same OD_600_, 200 µl of each suspension were centrifuged and the pellets resuspended in protein sample buffer. This was done in order to obtain the same amount of protein in all the samples analyzed. For the swarming analysis, cells were sampled from the center or from the border of the colony of swarming plates (SB-0.7% agar) and bacteria were recovered with PBS and standardized as described above for liquid culture bacteria. Equal volumes (20 µl) of these whole-cell extracts were loaded onto 12% SDS-PAGE gels and transferred to nitrocellulose membranes. Flagella were stained according to [49]. Stain was prepared by mixing 10 parts mordant (2 g tannic acid, 10 ml 5% phenol, 10 ml saturated aqueous AlKO_8_S_2_.12 H_2_O) with 1 part stain (12% crystal violet in ethanol). Bacteria were recovered from the center or the border of swarm plates with PBS and 20 µl of the suspensions were applied to a microscope slide and covered with a coverslip. The slide was propped vertically and 50 µl of dye was applied to the top edge of the coverslip to stain the sample by capillary action. Samples were observed with phase contrast using a Nikon Eclipse E800 microscope.

### Quantification of xanthan production

The quantification of xanthan production was done as described previously [50]. Briefly, strains were cultured to the same OD_600_ at 28°C in 50 ml of XOL modified liquid medium supplemented with 1% (w/v) glucose in 250 ml flasks, using an orbital shaker rotating at 200 rpm. At different times, the cells were removed by centrifugation (25000 *g* for 30 min), the supernatant fluids were supplemented with KCl at 1% (w/v) final concentration and 2 vol ethanol were added. The precipitated crude xanthan was collected, dried and weighed. Triplicate measurements of xanthan production were made and each data point showed in the figure is an average of 5 independent experiments. Error bars indicate the standard error (p<0.01).
